# Reply: Axillary recurrences after sentinel node (SLN) biopsy without complete axillary dissection in breast cancer patients

**DOI:** 10.1038/sj.bjc.6602431

**Published:** 2005-03-01

**Authors:** R Reitsamer, F Peintinger

**Affiliations:** 1 Department of Senology, University Hospital Salzburg, Paracelsus Private Medical School Salzburg, Muellner Hauptstrasse 48, 5020 Salzburg, Austria; 2 Department of Obstetrics and Gynecology, General Hospital Leoben, Vordernbergerstrasse 42, 8700 Leoben, Austria

**Sir**,

The letter from Kristen *et al* summarizes and confirms what we wanted to communicate in this article. We are convinced that sentinel node biopsy (SLNB) in experienced hands is a safe and reliable method for axillary staging in a defined group of breast cancer patients. But on the other hand, there are some aspects in the SLNB technique that are not yet quite clear and well understood. One of the questions is with regard to the pathological assessment of SLNs. There are no standards on the number of sections or the distance of sections of the SLN. We know that we normally cannot miss a micrometastasis if we cut the SLN in serial sections with distances less than 200 *μ*m. But what is the impact of tumour cell emboli or single tumour cells in SLNs? What is the role of immunohistochemistry or rPCR in the identification of tumour cells? When is a negative SLN truly negative? We have no answers for these questions and there are no accepted standards for the pathological examination of SLNs. We have a lot of data for identification rates of SLNs and a lot of data for false-negative rates. We have less data for local or axillary recurrence-free survival, and no data for disease-free survival and overall survival of patients with SLNB alone in SLN-negative patients. There are ongoing trials, which will answer these questions, and from the scientific point of view, we should await the results of these trials before we recommend the replacement of a standard treatment procedure such as ALND by an innovative method such as SLNB. Until we obtain these data, patients have to be informed about the status of SLNB and patients should have the choice of deciding between both treatment procedures. If we have evidence-based data for the superiority of SLNB compared to ALND and if we have standards for performing this fascinating technique, SLNB will be appreciated as a standard procedure for axillary lymph node-negative patients.

